# 3-Benzyl-6-bromo-2-(2-fur­yl)-3*H*-imidazo[4,5-*b*]pyridine

**DOI:** 10.1107/S160053681002475X

**Published:** 2010-06-30

**Authors:** Younès Ouzidan, Youssef Kandri Rodi, Hafid Zouihri, El Mokhtar Essassi, Seik Weng Ng

**Affiliations:** a Laboratoire de Chimie Organique Appliquée, Faculté des Sciences et Techniques, Université Sidi Mohamed Ben Abdallah, Fès, Morocco; b CNRST Division UATRS, Angle Allal Fassi/FAR, BP 8027 Hay Riad, Rabat, Morocco; c Laboratoire de Chimie Organique Hétérocyclique, Pôle de Compétences Pharmacochimie, Université Mohammed V-Agdal, BP 1014 Avenue Ibn Batout, Rabat, Morocco; d Department of Chemistry, University of Malaya, 50603 Kuala Lumpur, Malaysia

## Abstract

In the title mol­ecule, C_17_H_12_BrN_3_O, the imidazopyridine ring system is almost coplanar with the furan ring [dihedral angle = 2.0 (3)°]. The benzyl phenyl ring is oriented at dihedral angles of 85.2 (2) and 85.5 (1)°, respectively, with respect to the furan ring and the imidazopyridine ring system. In the crystal, mol­ecules are linked into chains propagating along the *b* axis by C—H⋯N hydrogen bonds. Adjacent chains are linked *via* short Br⋯Br contacts [3.493 (1) Å].

## Related literature

For a related structure, see: Ouzidan *et al.* (2010 ▶).
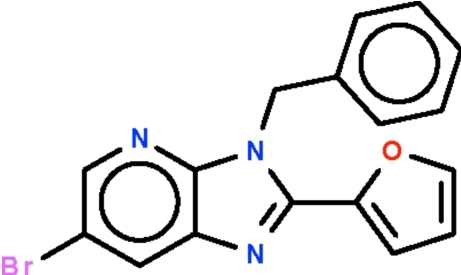


## Experimental

### 

#### Crystal data

C_17_H_12_BrN_3_O
*M* *_r_* = 354.21
Monoclinic, 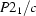

*a* = 15.8422 (3) Å
*b* = 5.4747 (1) Å
*c* = 18.4243 (3) Å
β = 111.509 (1)°
*V* = 1486.68 (5) Å^3^
*Z* = 4
Mo *K*α radiation
μ = 2.77 mm^−1^
*T* = 293 K
0.25 × 0.25 × 0.10 mm

#### Data collection

Bruker X8 APEXII area-detector diffractometer
Absorption correction: multi-scan (*SADABS*; Sheldrick, 1996 ▶) *T*_min_ = 0.544, *T*_max_ = 0.769
19471 measured reflections
2614 independent reflections
2105 reflections with *I* > 2σ(*I*)
*R*_int_ = 0.036

#### Refinement

*R*[*F* ^2^ > 2σ(*F* ^2^)] = 0.031
*wR*(*F* ^2^) = 0.110
*S* = 0.97
2614 reflections
199 parameters
H-atom parameters constrained
Δρ_max_ = 0.33 e Å^−3^
Δρ_min_ = −0.44 e Å^−3^

### 

Data collection: *APEX2* (Bruker, 2008 ▶); cell refinement: *SAINT* (Bruker, 2008 ▶); data reduction: *SAINT*; program(s) used to solve structure: *SHELXS97* (Sheldrick, 2008 ▶); program(s) used to refine structure: *SHELXL97* (Sheldrick, 2008 ▶); molecular graphics: *X-SEED* (Barbour, 2001 ▶); software used to prepare material for publication: *publCIF* (Westrip, 2010 ▶).

## Supplementary Material

Crystal structure: contains datablocks global, I. DOI: 10.1107/S160053681002475X/ci5113sup1.cif

Structure factors: contains datablocks I. DOI: 10.1107/S160053681002475X/ci5113Isup2.hkl

Additional supplementary materials:  crystallographic information; 3D view; checkCIF report

## Figures and Tables

**Table 1:** Hydrogen-bond geometry (Å, °)

*D*—H⋯*A*	*D*—H	H⋯*A*	*D*⋯*A*	*D*—H⋯*A*
C3—H3⋯N3^i^	0.93	2.51	3.399 (4)	160

Symmetry code: (i) 
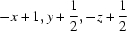
.

**Fig. 1.**
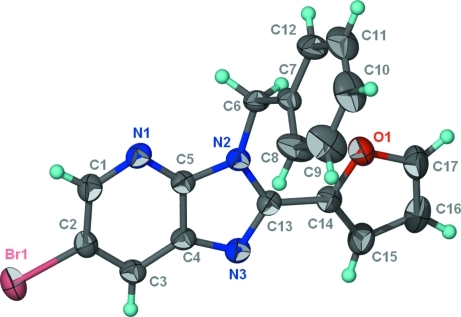
Displacement ellipsoid plot (Barbour, 2001) of the molecule of C17H12BrN3O at the 50% probability level. H atoms are shown as spheres of arbitrary radii.

## supplementary crystallographic information

### Comment

The imidazo[4,5-*b*]pyridine unit is an important heterocyclic nucleus found in a large number of molecules in medicinal chemistry. Heterocycles derived from such compounds posess useful medicinal properties. Owing to their importance, strategies have been developed for their synthesis. The most popular synthetic approach involves the cyclocondensation of 2,3-pyridinediamine with carboxylic acid derivatives or on condensation with aldehydes. An earlier study reported the crystal structure of 4-benzyl-6-bromo-2-phenyl-4*H*-imidazo[4,5-*b*]pyridine (Ouzidan *et al.*, 2010), which was synthesized by using a much more convenient route. The synthesis is extended to the title compound.

In the title molecule (Scheme and Fig. 1), the imidazopyridine ring system is almost coplanar with the furan ring at the 2-position of the five-membered ring [dihedral angle = 2.0 (3) °]. The molecules are linked into chains along the *b* axis by C—H···N hydrogen bonds (Table 1). The adjacent chains are linked via short Br···Br contacts [3.493 (1) Å].

### Experimental

6-Bromo-2-furyl-3*H*-imidazo[4,5-*b*]pyridine (0.30 g, 1.13 mmol) was dissolved in DMF (15 ml). Potassium carbonate (0.2 g, 1,48 mmol), tetra-*n*-butylammonium bromide (0.04 g, 0.1 mmol) and benzyl chloride (0.15 ml, 1.36 mmol) were added. Stirring was continued at room temperature for 12 h. The mixture was filtered and the solvent removed under reduced pressure. The residue was chromatographed on a column of silica gel with ethyl acetate-hexane (1/2) as eluent. The compound was recrystallized from chloroform to give orange crystals.

### Refinement

H atoms were placed in calculated positions (C–H = 0.93–0.97 Å) and were included in the refinement in the riding model approximation, with *U*(H) set to 1.2*U*_eq_(C).

### Crystal data

**Table:**

C_17_H_12_BrN_3_O	*F*(000) = 712
*M**_r_* = 354.21	*D*_x_ = 1.583 Mg m^−^^3^
Monoclinic, *P*2_1_/*c*	Mo *K*α radiation, λ = 0.71073 Å
Hall symbol: -P 2ybc	Cell parameters from 5830 reflections
*a* = 15.8422 (3) Å	θ = 2.7–23.3°
*b* = 5.4747 (1) Å	µ = 2.77 mm^−^^1^
*c* = 18.4243 (3) Å	*T* = 293 K
β = 111.509 (1)°	Prism, orange
*V* = 1486.68 (5) Å^3^	0.25 × 0.25 × 0.10 mm
*Z* = 4	

### Data collection

**Table:**

Bruker X8 APEXII area-detector diffractometer	2614 independent reflections
Radiation source: fine-focus sealed tube	2105 reflections with *I* > 2σ(*I*)
graphite	*R*_int_ = 0.036
φ and ω scans	θ_max_ = 25.0°, θ_min_ = 2.8°
Absorption correction: multi-scan (*SADABS*; Sheldrick, 1996)	*h* = −17→18
*T*_min_ = 0.544, *T*_max_ = 0.769	*k* = −6→5
19471 measured reflections	*l* = −21→18

### Refinement

**Table:**

Refinement on *F*^2^	Primary atom site location: structure-invariant direct methods
Least-squares matrix: full	Secondary atom site location: difference Fourier map
*R*[*F*^2^ > 2σ(*F*^2^)] = 0.031	Hydrogen site location: inferred from neighbouring sites
*wR*(*F*^2^) = 0.110	H-atom parameters constrained
*S* = 0.97	*w* = 1/[σ^2^(*F*_o_^2^) + (0.0848*P*)^2^ + 0.0891*P*] where *P* = (*F*_o_^2^ + 2*F*_c_^2^)/3
2614 reflections	(Δ/σ)_max_ = 0.001
199 parameters	Δρ_max_ = 0.33 e Å^−^^3^
0 restraints	Δρ_min_ = −0.44 e Å^−^^3^

### Fractional atomic coordinates and isotropic or equivalent isotropic displacement parameters (Å^2^)

**Table:**

	*x*	*y*	*z*	*U*_iso_*/*U*_eq_	
Br1	0.47189 (2)	1.27997 (6)	0.426257 (19)	0.05859 (18)	
O1	0.21672 (14)	0.1051 (4)	0.11117 (12)	0.0605 (6)	
N1	0.28026 (15)	0.7379 (4)	0.34628 (14)	0.0407 (6)	
N2	0.26035 (13)	0.4610 (4)	0.23929 (12)	0.0362 (5)	
N3	0.37245 (14)	0.6006 (4)	0.20155 (13)	0.0415 (5)	
C1	0.33253 (18)	0.9234 (5)	0.38466 (16)	0.0437 (6)	
H1	0.3214	0.9916	0.4265	0.052*	
C2	0.40263 (16)	1.0193 (5)	0.36533 (15)	0.0399 (6)	
C3	0.42374 (17)	0.9297 (5)	0.30393 (15)	0.0400 (6)	
H3	0.4701	0.9949	0.2904	0.048*	
C4	0.37115 (19)	0.7370 (4)	0.26428 (18)	0.0364 (6)	
C5	0.30126 (16)	0.6536 (5)	0.28810 (15)	0.0347 (6)	
C6	0.18408 (17)	0.3215 (5)	0.24445 (16)	0.0398 (6)	
H6A	0.1851	0.3335	0.2973	0.048*	
H6B	0.1923	0.1509	0.2344	0.048*	
C7	0.09249 (16)	0.4032 (4)	0.18891 (15)	0.0373 (6)	
C8	0.0798 (2)	0.6091 (6)	0.1437 (2)	0.0660 (9)	
H8	0.1294	0.7050	0.1467	0.079*	
C9	−0.0063 (3)	0.6757 (7)	0.0935 (3)	0.0863 (13)	
H9	−0.0139	0.8138	0.0623	0.104*	
C10	−0.0799 (2)	0.5399 (7)	0.0897 (2)	0.0752 (10)	
H10	−0.1375	0.5839	0.0556	0.090*	
C11	−0.0687 (2)	0.3409 (8)	0.1356 (2)	0.0706 (10)	
H11	−0.1190	0.2505	0.1340	0.085*	
C12	0.0171 (2)	0.2703 (5)	0.1851 (2)	0.0549 (8)	
H12	0.0239	0.1319	0.2160	0.066*	
C13	0.30648 (16)	0.4385 (5)	0.18846 (15)	0.0369 (6)	
C14	0.2871 (2)	0.2623 (4)	0.12615 (18)	0.0428 (7)	
C15	0.3289 (2)	0.2287 (6)	0.0754 (2)	0.0587 (9)	
H15	0.3787	0.3147	0.0737	0.070*	
C16	0.2825 (2)	0.0376 (6)	0.02513 (19)	0.0651 (9)	
H16	0.2956	−0.0266	−0.0163	0.078*	
C17	0.2169 (3)	−0.0318 (6)	0.04850 (19)	0.0683 (9)	
H17	0.1761	−0.1570	0.0257	0.082*	

### Atomic displacement parameters (Å^2^)

**Table:**

	*U*^11^	*U*^22^	*U*^33^	*U*^12^	*U*^13^	*U*^23^
Br1	0.0540 (3)	0.0519 (2)	0.0593 (3)	−0.00676 (12)	0.00831 (18)	−0.01181 (13)
O1	0.0659 (14)	0.0602 (13)	0.0594 (14)	−0.0183 (11)	0.0276 (11)	−0.0162 (10)
N1	0.0330 (13)	0.0534 (13)	0.0395 (14)	0.0029 (9)	0.0176 (11)	−0.0003 (10)
N2	0.0268 (11)	0.0449 (11)	0.0378 (13)	−0.0025 (9)	0.0130 (9)	−0.0003 (9)
N3	0.0317 (12)	0.0538 (14)	0.0424 (13)	−0.0020 (10)	0.0175 (10)	−0.0037 (10)
C1	0.0380 (15)	0.0519 (15)	0.0416 (16)	0.0036 (12)	0.0150 (12)	−0.0047 (12)
C2	0.0330 (14)	0.0416 (13)	0.0391 (16)	0.0017 (11)	0.0061 (12)	−0.0006 (11)
C3	0.0296 (14)	0.0464 (14)	0.0440 (16)	−0.0006 (11)	0.0135 (12)	0.0030 (11)
C4	0.0281 (15)	0.0436 (14)	0.0396 (17)	0.0007 (10)	0.0147 (13)	0.0028 (11)
C5	0.0253 (13)	0.0419 (13)	0.0361 (15)	0.0025 (10)	0.0101 (11)	0.0027 (11)
C6	0.0345 (15)	0.0458 (14)	0.0414 (16)	−0.0046 (11)	0.0165 (13)	0.0047 (11)
C7	0.0288 (13)	0.0408 (13)	0.0437 (16)	−0.0052 (10)	0.0152 (12)	−0.0047 (11)
C8	0.0388 (17)	0.063 (2)	0.086 (2)	−0.0040 (14)	0.0108 (17)	0.0219 (17)
C9	0.062 (2)	0.069 (2)	0.102 (3)	0.0088 (19)	−0.001 (2)	0.025 (2)
C10	0.0368 (19)	0.089 (3)	0.083 (3)	0.0077 (17)	0.0013 (17)	−0.012 (2)
C11	0.0350 (19)	0.093 (3)	0.081 (3)	−0.0156 (17)	0.0181 (19)	−0.015 (2)
C12	0.0417 (19)	0.0643 (19)	0.061 (2)	−0.0119 (13)	0.0216 (16)	0.0033 (14)
C13	0.0291 (13)	0.0446 (13)	0.0366 (15)	0.0046 (10)	0.0116 (11)	0.0011 (11)
C14	0.0353 (16)	0.0481 (15)	0.0427 (18)	0.0018 (11)	0.0117 (13)	−0.0002 (11)
C15	0.053 (2)	0.074 (2)	0.054 (2)	−0.0036 (14)	0.0244 (18)	−0.0152 (15)
C16	0.076 (2)	0.070 (2)	0.049 (2)	0.0112 (18)	0.0221 (17)	−0.0105 (15)
C17	0.091 (3)	0.0550 (18)	0.054 (2)	−0.0113 (18)	0.0199 (19)	−0.0155 (15)

### Geometric parameters (Å, °)

**Table:**

Br1—C2	1.897 (3)	C7—C8	1.372 (4)
O1—C14	1.355 (3)	C7—C12	1.377 (4)
O1—C17	1.378 (4)	C8—C9	1.387 (5)
N1—C5	1.317 (3)	C8—H8	0.93
N1—C1	1.337 (3)	C9—C10	1.362 (5)
N2—C5	1.383 (3)	C9—H9	0.93
N2—C13	1.388 (3)	C10—C11	1.351 (5)
N2—C6	1.462 (3)	C10—H10	0.93
N3—C13	1.324 (3)	C11—C12	1.385 (5)
N3—C4	1.382 (4)	C11—H11	0.93
C1—C2	1.388 (4)	C12—H12	0.93
C1—H1	0.93	C13—C14	1.444 (4)
C2—C3	1.382 (4)	C14—C15	1.341 (5)
C3—C4	1.376 (4)	C15—C16	1.412 (4)
C3—H3	0.93	C15—H15	0.93
C4—C5	1.408 (4)	C16—C17	1.317 (5)
C6—C7	1.505 (4)	C16—H16	0.93
C6—H6A	0.97	C17—H17	0.93
C6—H6B	0.97		
			
C14—O1—C17	105.2 (2)	C7—C8—C9	120.7 (3)
C5—N1—C1	113.9 (2)	C7—C8—H8	119.7
C5—N2—C13	105.62 (19)	C9—C8—H8	119.7
C5—N2—C6	123.9 (2)	C10—C9—C8	120.3 (4)
C13—N2—C6	130.5 (2)	C10—C9—H9	119.8
C13—N3—C4	105.2 (2)	C8—C9—H9	119.8
N1—C1—C2	123.4 (3)	C11—C10—C9	119.6 (3)
N1—C1—H1	118.3	C11—C10—H10	120.2
C2—C1—H1	118.3	C9—C10—H10	120.2
C3—C2—C1	122.2 (2)	C10—C11—C12	120.6 (3)
C3—C2—Br1	119.38 (19)	C10—C11—H11	119.7
C1—C2—Br1	118.46 (19)	C12—C11—H11	119.7
C4—C3—C2	115.2 (2)	C7—C12—C11	120.6 (3)
C4—C3—H3	122.4	C7—C12—H12	119.7
C2—C3—H3	122.4	C11—C12—H12	119.7
C3—C4—N3	131.8 (2)	N3—C13—N2	113.1 (2)
C3—C4—C5	118.4 (3)	N3—C13—C14	121.1 (2)
N3—C4—C5	109.9 (2)	N2—C13—C14	125.8 (2)
N1—C5—N2	126.8 (2)	C15—C14—O1	110.5 (3)
N1—C5—C4	127.0 (2)	C15—C14—C13	129.1 (3)
N2—C5—C4	106.2 (2)	O1—C14—C13	120.3 (3)
N2—C6—C7	114.5 (2)	C14—C15—C16	106.7 (3)
N2—C6—H6A	108.6	C14—C15—H15	126.6
C7—C6—H6A	108.6	C16—C15—H15	126.6
N2—C6—H6B	108.6	C17—C16—C15	106.4 (3)
C7—C6—H6B	108.6	C17—C16—H16	126.8
H6A—C6—H6B	107.6	C15—C16—H16	126.8
C8—C7—C12	118.1 (3)	C16—C17—O1	111.2 (3)
C8—C7—C6	123.2 (2)	C16—C17—H17	124.4
C12—C7—C6	118.6 (2)	O1—C17—H17	124.4
			
C5—N1—C1—C2	0.0 (4)	C6—C7—C8—C9	−180.0 (3)
N1—C1—C2—C3	0.2 (4)	C7—C8—C9—C10	1.4 (7)
N1—C1—C2—Br1	−179.2 (2)	C8—C9—C10—C11	0.7 (7)
C1—C2—C3—C4	−0.8 (4)	C9—C10—C11—C12	−1.7 (6)
Br1—C2—C3—C4	178.56 (19)	C8—C7—C12—C11	1.3 (5)
C2—C3—C4—N3	−179.3 (3)	C6—C7—C12—C11	179.1 (3)
C2—C3—C4—C5	1.2 (4)	C10—C11—C12—C7	0.7 (6)
C13—N3—C4—C3	179.7 (3)	C4—N3—C13—N2	0.5 (3)
C13—N3—C4—C5	−0.7 (3)	C4—N3—C13—C14	179.9 (2)
C1—N1—C5—N2	178.7 (2)	C5—N2—C13—N3	0.0 (3)
C1—N1—C5—C4	0.4 (4)	C6—N2—C13—N3	179.7 (2)
C13—N2—C5—N1	−179.0 (2)	C5—N2—C13—C14	−179.4 (2)
C6—N2—C5—N1	1.3 (4)	C6—N2—C13—C14	0.3 (4)
C13—N2—C5—C4	−0.4 (3)	C17—O1—C14—C15	0.7 (4)
C6—N2—C5—C4	179.9 (2)	C17—O1—C14—C13	179.1 (3)
C3—C4—C5—N1	−1.1 (4)	N3—C13—C14—C15	1.2 (5)
N3—C4—C5—N1	179.3 (2)	N2—C13—C14—C15	−179.5 (3)
C3—C4—C5—N2	−179.7 (2)	N3—C13—C14—O1	−176.9 (2)
N3—C4—C5—N2	0.7 (3)	N2—C13—C14—O1	2.4 (4)
C5—N2—C6—C7	96.8 (3)	O1—C14—C15—C16	−0.3 (4)
C13—N2—C6—C7	−82.8 (3)	C13—C14—C15—C16	−178.5 (3)
N2—C6—C7—C8	−7.5 (4)	C14—C15—C16—C17	−0.3 (4)
N2—C6—C7—C12	174.8 (2)	C15—C16—C17—O1	0.7 (4)
C12—C7—C8—C9	−2.3 (5)	C14—O1—C17—C16	−0.8 (4)

### Hydrogen-bond geometry (Å, °)

**Table:**

*D*—H···*A*	*D*—H	H···*A*	*D*···*A*	*D*—H···*A*
C3—H3···N3^i^	0.93	2.51	3.399 (4)	160

Symmetry codes: (i) −*x*+1, *y*+1/2, −*z*+1/2.
